# Remarks on the Tread-Wheel

**Published:** 1823-10

**Authors:** J. C. Hippisley

**Affiliations:** Bart. &c. &c.


ART. III.—*Remarks on the Tread-Wheel.* Contained in a Letter from Sir J. C. HIPPISLEY, Bart. &c. &c. to the Editors.

A LETTER, addressed to the " Visiting Justices of the Nottinghamshire House of Correction," under the signature of Mr. Hutchinson, surgeon of the establishment, having appeared in your last Journal, with some observations on a communication which I thought it my duty to make, generally, to the magistracy in the course of the last year, I cannot but notice that, when Mr. Hutchinson speaks of " the accident on the machinery by which the *boys* at work on another wheel (in the House of Correction in Cold-bath Fields,) had been miserably crushed," he merely observes, " I can only assert that accidents of this description have not happened in the *Nottinghamshire* House of Correction; neither do I see *any probability of their occurrence*, excepting by the *extreme carelessness* of those on the wheel."

If Mr. Hutchinson had considered the incident deserving his notice at all, I should have expected from his candour that he would not have withheld the facts stated in the first page of the circular communication upon which he has commented. He must have observed that, with reference to the Official Returns of the Visiting Magistrates, printed by order of the House of Commons, " the question was put why, may it not be asked, are the various accidents, of the most serious complexion, passed over without the slightest notice whatever, that, in *different prisons*, have followed the use of the tread-machinery,—particularly those that have occurred from the breaking the shaft, or continuation of the axis, *four times in the prison of Cold-bath Fields?* Nor has the shaft at Brixton escaped a similar lot: and, without the influence of such a bias (as has been noticed), it would be inconceivable that a report which correctly states that the tread-mill discipline was applied to *females* in the former prison, should be silent as to the chief motives for its discontinuance."*

I will not trespass upon your pages with the repetition of details which are now pretty generally before the public. Having myself made an unsuccessful attempt to resist the introduction of this novel, and as I conceive injurious, discipline, in a considerable provincial establishment, where for many years I had acted as a visiting magistrate, it is, under the present circumstances, most consistent with my feelings to request the discontinuance of my appointment to that highly responsible office. I have also thought it no less my duty to submit to the

* When this observation was made, the female prisoners had been withdrawn from the discipline, since which period it has been unfortunately resumed.

public the communications which have now become subjects of much discussion. I can accuse myself for withholding two letters only, from professional gentlemen whose opinions differ from those which constitute the greater part of the correspondence, which has extended to so great a length.

I will only further observe, that I conceive all the material observations of Mr. Hutchinson are fully answered, by *anticipation*, in Dr. Good's letter of the 7th of June last. That letter, with some additional notes, has been sent to the press for a reprint; and, should any advantage result from the sale, or of that of the more extended correspondence, it will be applied to the general benevolent purposes of the Society for the Improvement in Prison Discipline. Of Dr. Good's professional merits, Mr. Hutchinson has spoken very handsomely.

I have availed myself of a visit of Mr. Cole to his family, residing in this county, to request his attestation to the several facts which occurred in his repeated visits, with Dr. Good and myself, to the prison in Cold-bath Fields; since which other casualties of the same description have occurred, though "Mr. Hutchinson saw no probability of their recurrence, except by the *extreme carelessness* of those on the wheel." My own opinion, nevertheless, is so much at variance with that of Mr. Hutchinson, that I am fully persuaded that it is not within the means of Mr. Cubitt's efforts to effect any *adequate guard* against their recurrence.

I am, Gentlemen, very obediently your's,

J. C. HIPPISLEY.

*West Cowes, Isle of Wight; 29th Aug.* 1823.

*Letter of* Mr. Cole, *Surgeon to the Northern Dispensary, to* Sir J. C. Hippisley, Bart.

Dear Sir,—I have no hesitation in answering your question as to "the accuracy of Dr. Good's statement of the facts and observations on those occasions when I visited with him the House of Correction in Cold-bath Fields:" nor have I any difficulty in avowing my opinions on the subject, as a professional man, being in entire concurrence with those of Dr. Good.

During my visit to my family connexions in this place, I have taken an opportunity of viewing the tread-wheel in the prison at Newport, where it is admitted that casualties, similar to those which, in a greater extent, have taken place in the prison at Cold-bath Fields, (as described by yourself and Dr. Good,) have also occurred: both men and women have here frequently fallen from the wheel; and one man recently received a fall with much injury, which confined him near two months under the care of the medical attendant.

The remonstrances of the *women* against the discipline have been so powerfully urged, even against the strongest menaces of the keeper, that he has been obliged to forego the practice of putting them on the wheel.

The keeper observed, that the discipline was so severe that it deterred many who otherwise would have submitted to the imprisonment, rather than pay the fines which exempted them from it. If such be the fact, I need make no comment to you, Sir, who have so perseveringly investigated the subject, on the inconsistency of such inflictions, as well as the inequality of punishment, which is made dependent, in a certain degree, on the pocket rather than on the delinquency of the offender.

I have the honour to remain, dear Sir, yours, &c. &c.

ROBERT COLE.

**To Sir J. C. Hippisley, Bart.**

***West Cowes; 28th August,* 1823.**

